# Molecular mechanisms of herbal medicines and bioactive compounds in rheumatoid arthritis: a comprehensive network pharmacology perspective

**DOI:** 10.1007/s11033-026-12727-5

**Published:** 2026-09-10

**Authors:** T. J. Jasna, K. S. Sandra, Megh Pravin Vithalkar, D. Vijaykumar, Yogendra Nayak

**Affiliations:** 1 Department of Pharmacology, Manipal College of Pharmaceutical Sciences, Manipal Academy of Higher Education, Manipal, India; 2 Department of Pharmacognosy, Nehru College of Pharmacy, Thrissur, Kerala India; 3 Department of Pharmaceutical Chemistry, Nehru College of Pharmacy, Thrissur, Kerala India

**Keywords:** Rheumatoid arthritis, Network pharmacology, Traditional medicines, Bioactive phytochemicals, Molecular pathways, Herbal drugs

## Abstract

Rheumatoid arthritis (RA) is a chronic immune-mediated inflammatory disease characterized by persistent synovial inflammation, progressive joint distruction, and systemic complications. Owing to the multifactorial nature of RA, therapies targeting a single molecular pathway often provide incomplete disease control and may be associated with long-term adverse effects. Network pharmacology has emerged as a powerful system-level approach that integrates bioinformatics, multi-omics datasets, molecular docking, and experimental validation to unravel the complex interactions among bioactive compounds, molecular targets, and signaling pathways. This review highlights the novel application of network pharmacology in deciphering the multi-target mechanisms of herbal medicines for RA and summarizes recent advances in this rapidly evolving field. Particular emphasis is placed on key pathogenic pathways, including NF-κB, PI3K/Akt, MAPK, JAK/STAT, NLRP3 inflammasome activation, oxidative stress responses, angiogenesis, and immune-cell dysregulation. Representative phytochemicals, such as quercetin, kaempferol, luteolin, triptolide, and berberine, are discussed for their ability to modulate inflammatory responses, fibroblast-like synoviocyte activation, macrophage polarization, and osteoclastogenesis through coordinated regulation of multiple signaling networks. Importantly, this review underscores the translational significance of network pharmacology as a bridge between traditional herbal medicine and modern drug discovery, facilitating target prioritization, mechanism-based validation, and the rational development of multi-target therapeutics for RA. Collectively, network pharmacology provides a robust framework for the mechanistic interpretation of herbal medicines and offers a promising opportunity to accelerate the discovery and clinical translation of safer, more effective therapies for RA.

**Graphical Abstract:**

**Figure:**
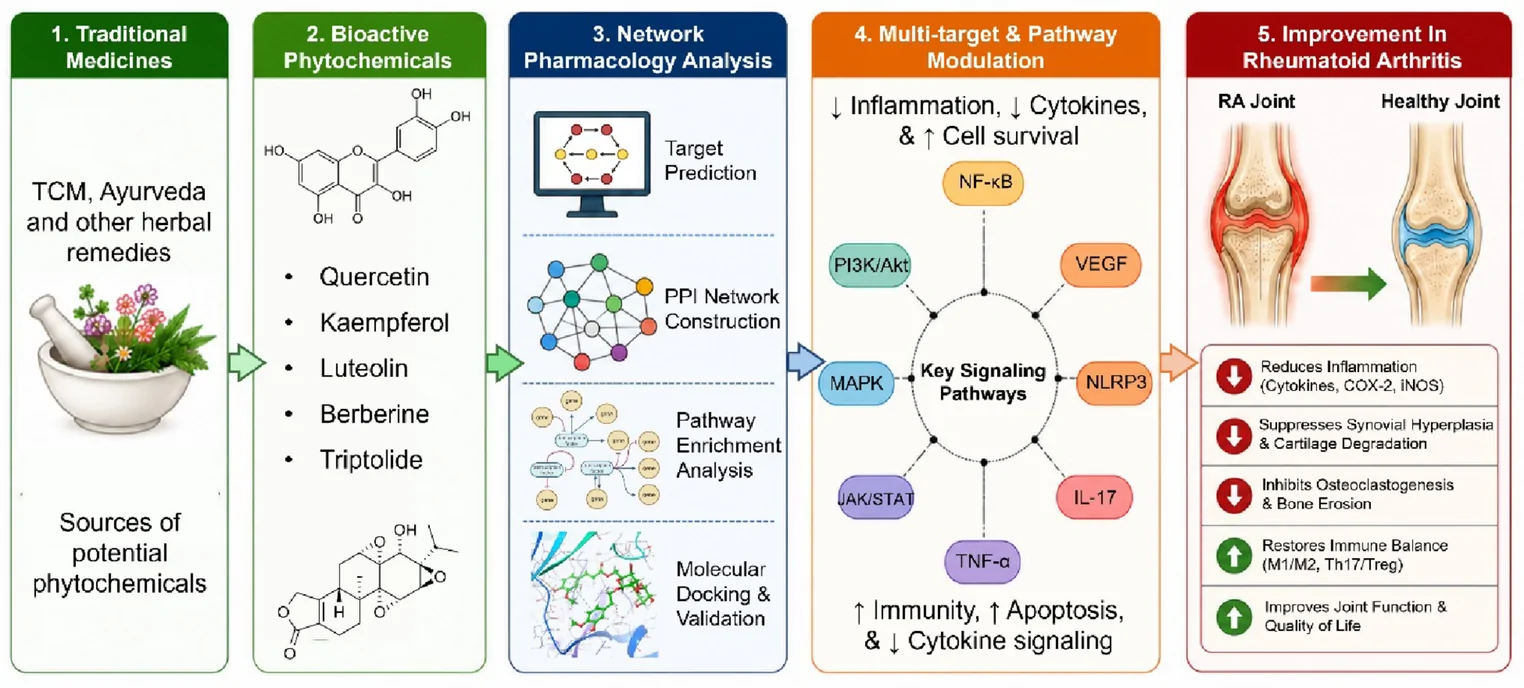

## Introduction

Rheumatoid arthritis (RA) is a chronic systemic autoimmune disease characterised by persistent synovial inflammation, progressive cartilage and bone destruction, joint deformity, and functional disability. Its pathogenesis involves complex interactions among inflammatory cytokines, immune cells, oxidative stress mediators, and dysregulated signaling pathways, including TNF-α, IL-1β, and IL-6, which collectively sustain synovial inflammation and tissue destruction [1]. Although genetic, environmental, hormonal, and lifestyle factors contribute to disease susceptibility, the precise etiology remains incompletely understood, underscoring the need for multi-target therapeutic strategies [2]. RA affects approximately 1% of the global population, with markedly higher prevalence in women than in men, imposing substantial clinical and socioeconomic burden [3]. Current RA management relies on NSAIDs, glucocorticoids, conventional disease-modifying antirheumatic drugs (DMARDs), and biological agents. Although these therapies effectively reduce inflammation and delay disease progression, they are frequently associated with incomplete responses, high costs, adverse effects, and loss of efficacy over long-term treatment [4, 5]. These limitations have stimulated growing interest in multi-target therapeutic strategies capable of simultaneously modulating multiple pathogenic pathways involved in RA.

The multicomponent and multitarget nature of herbal medicines poses a major challenge for conventional pharmacological approaches that typically investigate single compounds acting on individual molecular targets. Network pharmacology has emerged as an effective systems biology-based strategy that integrates bioinformatics, molecular docking, omics technologies, and pharmacological databases to elucidate the complex interactions among phytochemicals, molecular targets, signaling pathways, and disease networks [6]. This holistic approach is particularly well-suited for RA, a multifactorial disease driven by interconnected inflammatory and immune pathways, because it enables the systematic characterization of the synergistic mechanisms underlying traditional herbal medicines. Traditional medical systems, including Ayurveda and Traditional Chinese Medicine (TCM), have long employed polyherbal formulations for the management of RA. Although many of these formulations demonstrate promising therapeutic efficacy, their molecular mechanisms remain incompletely understood because of the complex interactions among numerous bioactive constituents. Network pharmacology provides an ideal framework for deciphering these complex mechanisms by linking herbal components with molecular targets and disease-associated signaling pathways. Therefore, this review aims to summarize recent advances in the application of network pharmacology for investigating herbal medicines used in RA. Particular emphasis is placed on the identification of bioactive compounds, molecular targets, key signaling pathways, and experimental validation strategies, while discussing the translational potential of network pharmacology for accelerating mechanism-based herbal drug discovery and multi-target therapeutic development in RA.

The graphical abstract summarizes the overall concept and scope of this review. It illustrates the progression from traditional medicinal systems, including Ayurveda and TCM, to the identification of bioactive phytochemicals and their investigation using network pharmacology approaches, including target prediction, multiple network construction, pathway enrichment analysis, and molecular docking. These integrated analyses help elucidate the multi-target mechanisms through which phytochemicals modulate key inflammatory and immune signaling pathways discussed in the following sections. Collectively, these mechanisms contribute to reduced inflammation, preservation of joint integrity, restoration of immune balance, and improved clinical outcomes in RA. This review was conducted through a comprehensive literature search using the electronic databases PubMed, Scopus, Web of Science, and Google Scholar. Publications published between January 2020 and April 2026 were systematically searched using combinations of the following keywords: “rheumatoid arthritis,” “network pharmacology,” “traditional medicine,” “Traditional Chinese Medicine,” “Ayurveda,” “herbal medicine,” “phytochemicals,” “molecular docking,” and “systems pharmacology.” Reference lists of relevant articles were also manually screened to identify additional eligible studies. Studies were included if they (i) investigated the application of network pharmacology to herbal or traditional medicine-based therapies for rheumatoid arthritis, (ii) reported molecular targets, signaling pathways, bioactive phytochemicals, molecular docking, omics analyses, or experimental validation relevant to RA, and (iii) were published as original research articles or high-quality review articles in English. Studies were excluded if they focused on non-herbal interventions, unrelated diseases, conference abstracts, editorials, commentaries, duplicate publications, or articles lacking sufficient mechanistic information relevant to the scope of this review. The selected literature was critically evaluated to summarize current advances in applying network pharmacology to identify bioactive compounds, molecular targets, signaling pathways, and the multi-target mechanisms underlying traditional medicine-derived therapeutics for rheumatoid arthritis.

## Network pharmacology and its utilization in RA

A new method, network pharmacology, was developed to address the drawbacks of drug discovery in the present day. It is about understanding the drugs’ ability to bind to and regulate multiple targets simultaneously. Network pharmacology uses computer programs and virtual high-throughput screening to shorten the time required for drug discovery. Furthermore, network pharmacology enables the thorough examination of the complex drug-target-disease relationship by combining systems biology, bioinformatics, cheminformatics, molecular docking, and computational pharmacology [7].

**Fig. 1:**
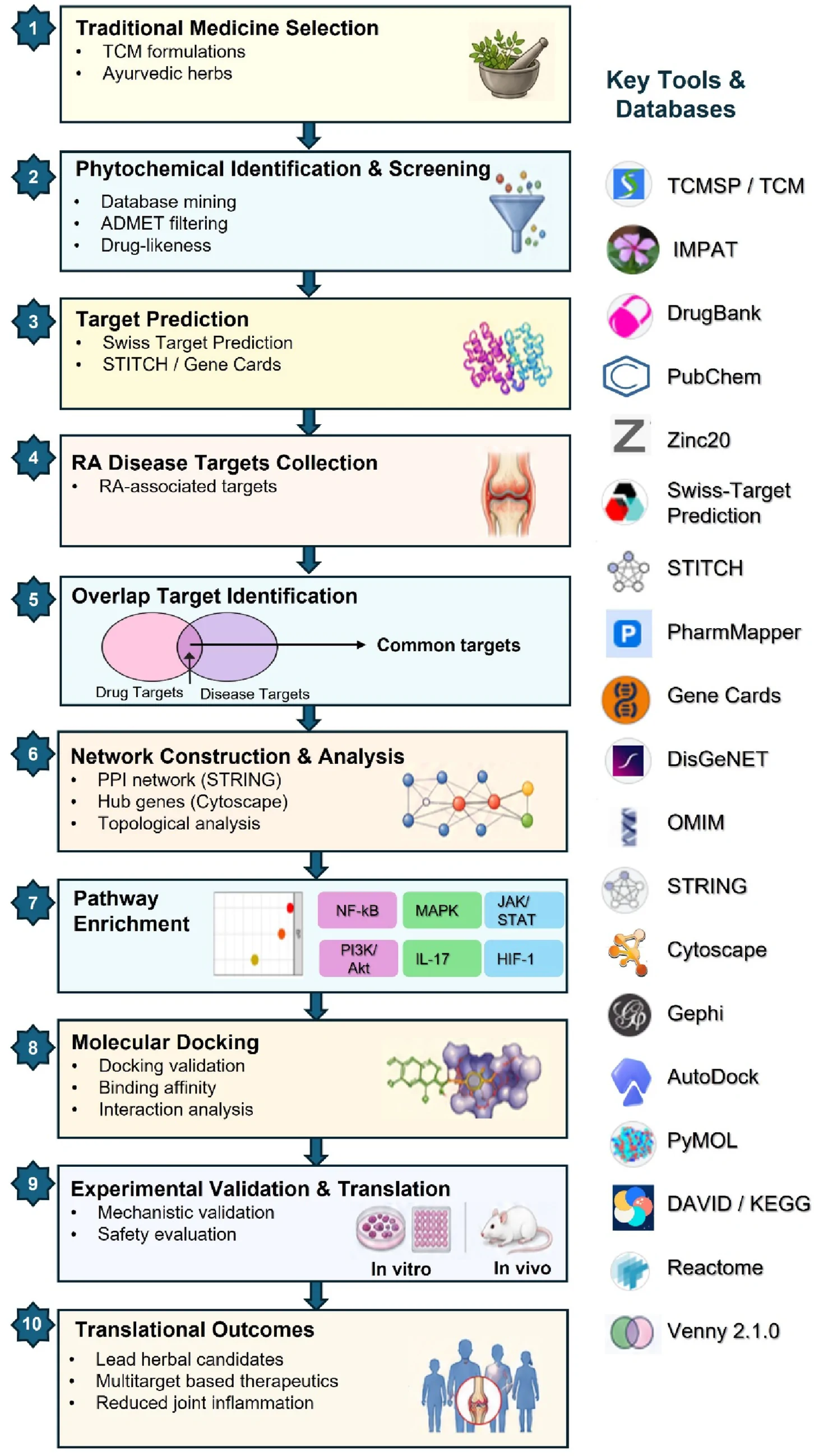
Network pharmacology-guided workflow for identifying traditional medicine-based therapeutics in rheumatoid arthritis. The figure illustrates the integrated workflow used in network pharmacology studies, including traditional medicine selection, phytochemical screening, target prediction, network and pathway analysis, molecular docking, and experimental validation, leading to the identification of multi-target therapeutic candidates for rheumatoid arthritis

Creating a detailed network model that links drugs, targets, and diseases enables scientists to explore how drugs could potentially treat a range of diseases. This development has taken pharmacological research from focusing on a single target to carrying out a comprehensive analysis of networks [8]. Most notably, despite the fact that direct experiments on humans are not feasible, network pharmacology can integrate knowledge about the human body with drug and disease databases to find correspondences. By producing diagrams of protein-to-protein interactions, performing molecular docking simulations, and utilizing other approaches, it can mirror the mechanisms of drugs in humans and discover new drug targets [9]. Network pharmacology is a great tool for shortlisting compounds or substances that may be capable of treating RA [10]. By generating interaction networks, researchers can predict the most likely drug targets. This gives them an opportunity to study the signaling pathways involved in RA. Furthermore, this technique helps in understanding drug mechanisms, discovering existing drugs that could be repurposed for RA treatment, and employing computer modeling and virtual experiments to evaluate the potential drug binding, predict drug efficacy, and examine the safety profiles of the lead compounds [11]. Figure 1 provides an overview of a combined workflow often utilized in network pharmacology research involving traditional herbal medicines. This illustration is meant to capture, step-wise, the analytical sequence involving phytochemical screening, target prediction, network building, pathway enrichment, molecular docking, and experimental verification. Also highlighted here, are the main databases as well as the different bioinformatics tools that are used throughout all stages of the drug discovery pipeline.

**Fig. 2:**
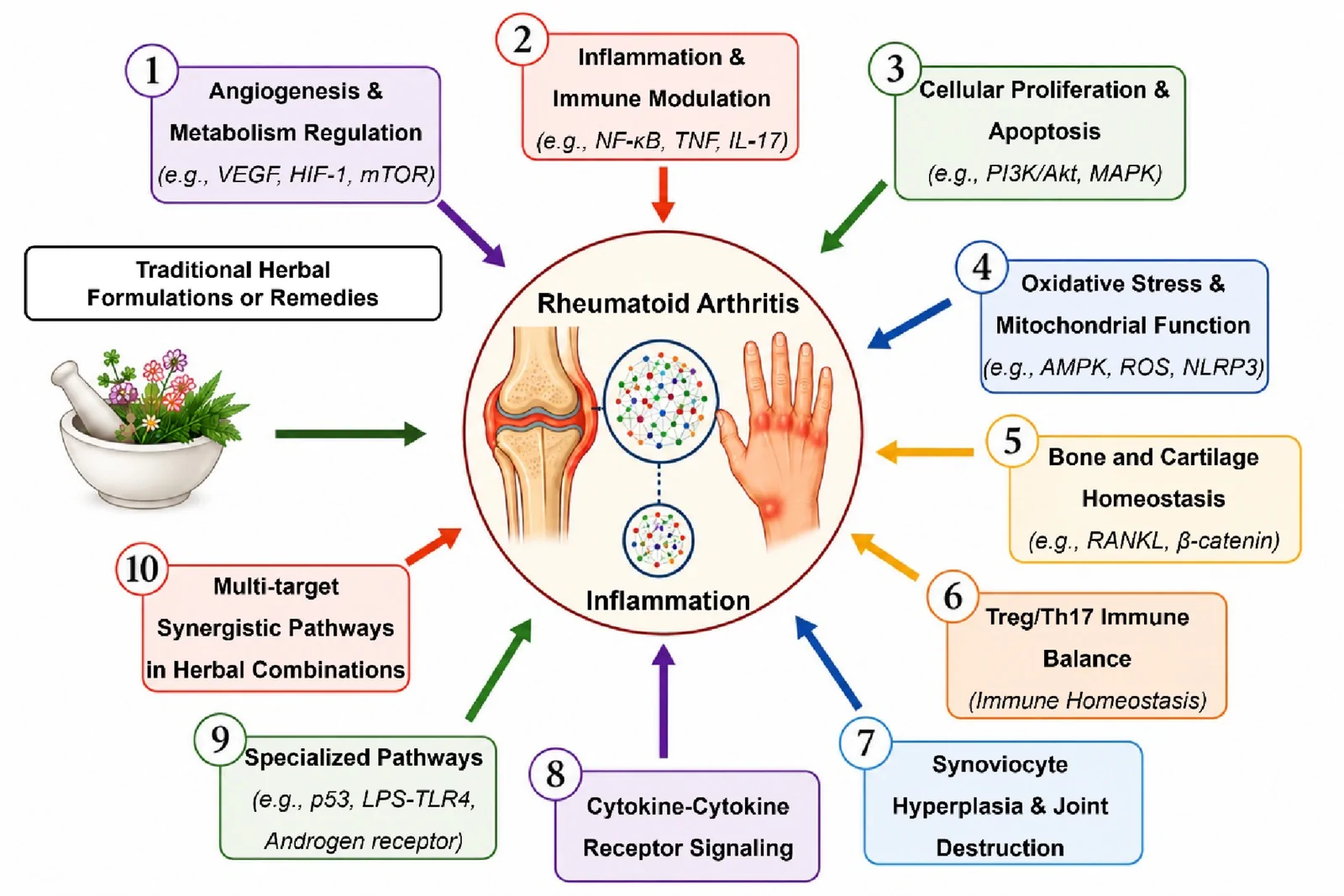
Major mechanistic clusters modulated by traditional medicines in rheumatoid arthritis. The figure summarizes key molecular pathways involved in inflammation, immune regulation, apoptosis, oxidative stress, angiogenesis, bone remodeling, cytokine signaling, and synovial hyperplasia associated with rheumatoid arthritis pathogenesis

Network pharmacology emerged as principle approach for investigating the mechanism of action of traditional medicine formulations, plant-derived bioactive compounds, and natural products with anti-inflammatory and immunomodulatory properties in RA. Figure 2 provides an overview of the ten major mechanistic clusters through which these therapies exert their therapeutic effects. These include the regulation of inflammation and immune responses, cellular proliferation and apoptosis, oxidative stress and mitochondrial function, bone and cartilage homeostasis, Treg/Th17 immune balance, cytokine–cytokine receptor signaling, specialized signaling pathways, angiogenesis and metabolic regulation, and the synergistic multi-target actions of herbal combinations. Together, these interconnected mechanisms illustrate how network pharmacology enables a systems-level understanding of the complex interactions between herbal medicines and RA pathogenesis, forming the conceptual framework for the subsequent sections of this review.

## Network pharmacology to predict the mechanisms of drugs in treating RA

### Inflammation and immune response

Inflammation is a very important protective mechanism of the body. It helps the body to fight off the infection and heal damaged tissues. Yet in the cases of the chronic type, for instance, RA, the process can actually get out of hand and result in permanent joint inflammation. Using immune-modulating drugs such as corticosteroids, NSAIDs, and, particularly, biologics has been very helpful in controlling overactive immune responses [12]. NF-κB pathways regulate many genes involved in inflammation and cell survival. Additionally, its prolonged activation is closely associated with RA. Likewise, the IL-17 pathway, supported by Th17 cells, mediates the release of pro-inflammatory mediators and is a major player in the autoimmune tissue damage seen in RA and related conditions [13]. The TNF pathway is one of the major systems that drives systemic inflammation. TNF-α, a cytokine produced mainly by macrophages, is a critical factor or ‘master regulator’ in the pathogenesis of the disease. Numerous studies have shown that blocking TNF is highly effective at mitigating cytokine-induced injury in RA [14]. The cascades of “T-cell and B-cell receptor signaling” come into play when the immune system needs to be activated tightly and specifically. When dysregulated, they lead to autoimmunity by failing to distinguish self from non-self [15]. The cytokine-cytokine receptor interaction network coordinates communication across immune cells. Its disruption promotes sustained inflammation, commonly seen in RA and related disorders [16].

*Tripterygii Radix* alleviates RA through anti-inflammatory effects by inhibiting TNF signaling and regulating AKT1, JUN, CASP3, TNFR1/2, IL-6, and TNF-α, with reduced RA- fibroblast-like synoviocytes (FLS) migration. The major constituent with therapeutic potential was identified as kaempferol. Other phytoconstituents in *Tripterygii Radix* include β-sitosterol, stigmasterol, triptolide, celastrol, wilforlide A, wilforine, wilforgine and wilfortrine [17]. *Chaenomelis Fructus*, also known as “Mu-Gua” in TCM, regulates key signaling pathways to exert anti-inflammatory effects in RA. It modulates IL-17, TNF, TLR, and NF-kB pathways, reducing inflammation and joint damage, thereby promising as an adjunctive therapy for RA management [18]. *Tripterygium hypoglaucum* (THH), a Chinese traditional medicine herb, exhibits anti-RA effects through 17 active ingredients, including triptolide and celastrol. Other active molecules are diterpenoids (tripterolide, triptonide and triptophenolide), triterpenoids (celastrol, wilforlide A, demethylzeylasteral, wilforic acid, and canophyllal), and alkaloids (sesquiterpene pyridine alkaloids (wilforine, wilforgine, wilfortrine, tripterine). Network pharmacology reveals PTGS2 as a key target gene, and Triptolide and celastrol exhibit strong binding to PTGS2. THH inhibits inflammation by inactivating TNF/NF-kB signaling pathways. This study provides evidence for the clinical efficacy of THH in treating RA [19].

*Cinnamomi ramulus* (CR), known as Guizhi in Chinese, relieves RA symptoms in CIA rat models. Its bioactive compounds act on TNF and IL-17 signaling pathways, effectively reducing inflammation and oxidative stress in vitro. The major active constituents are phenylpropanoids (cinnamaldehyde, cinnamic acid, 2-methoxycinnamic acid, cinnamyl alcohol), essential oils (eugenol, cinnamyl acetate, L-borneol, phellandrene, α-terpineol, β-caryophyllene), coumarin and flavonoids (quercetin, kaempferol, luteolin, taxifolin, procyanidins), triterpenoid saponins, and sterols such as β-sitosterol [20]. *Trachelospermum jasminoides*, which is a traditional plant in China, has the potential to relieve RA symptoms through blocking IL-17 and TNF signaling pathways. The herb modulates immune and inflammatory functions, and also demonstrates anti-anxiety, anti-depression, and estrogen-like effects. The lead molecules identified are trachelogenin, arctigenin, arctiin, 9-deoxycedrusin, ursolic acid, β-sitosterol, quercetin, luteolin, rutin, apigenin, and apigenin-7-O-glycosides, which have been reported to prevent chronic inflammation [21].

Myricaria germanica alleviates RA via key compounds apigenin, quillaic acid, and isorhamnetin. These compounds target TNF, PTGS2, and MMP9, regulating IL-17, TNF, and relaxin pathways [22]. Leocarpinolide B (LB), obtained from *Sigesbeckia Herba* (SH), a traditional Chinese medicinal herb, alleviates RA by inhibiting NF-κB p65 and reducing inflammation. LB suppresses the production of autoantibodies, cytokines, and synovial cell proliferation. By directly preventing NF-κB p65 from binding to DNA, LB disrupts the transcriptional regulation of inflammatory mediator genes. Results indicate that LB can be a potential bioactive agent that can be utilized in the development of new drugs for combating rheumatoid arthritis [23]. Similarly, calycosin, an isoflavonoid and phytoestrogen derived from the roots of *Astragalus membranaceus*, red clover (*Trifolium pratense*), Kudzu root (*Pueraria lobata*), or Black Cohosh (*Actaea racemosa*), alleviates RA symptoms by blocking JNK and NF-kB pathways. Calycosin suppresses synovial macrophage activation and tissue damage. This natural compound exhibits anti-inflammatory effects in CIA mice and macrophage models [24].

Huangqi Guizhi Wuwu Decoction (HQGZ), a formulation in TCM, treats RA via TNF signaling inhibition, with network pharmacology identifying 50 active components and 108 targets, and HQGZ regulates immune function, inflammation, and cell apoptosis by inhibiting key proteins like CASP3, TNF, RELA, and IKBKB in TNF-induced synoviocytes. The major therapeutic constituents can be calycosin, formononetin, astragalosides, paeoniflorin, albiflorin, benzoylpaeoniflorin, gingerols, shogaols, procyanidin B2, quercetin derivatives, triterpenoids, and cinnamaldehyde [25]. Er Miao San (EMS) treats RA by a multi-component, multi-target system of quercetin, wogonin, and rutaecarpine. These natural compounds suppress cytokine-induced inflammation, especially TNF-α, IL-6, and IL-13, while lowering nitric oxide levels and regulating major inflammatory signaling pathways. These various target effects highlight the potential of EMS as a treatment for RA. Experimental validation confirms anti-inflammatory effects of EMS, positioning it as a potential herbal drug for inflammatory and arthritic conditions. Major lead compounds include alkaloids such as berberine, phellodendrine, palmatine, and oxyberberine; flavonoids such as quercetin and wogonin; and volatile terpenoids such as atractylodin, atractylon, β-eudesmol, and atractylenolide I, II, and III [26].

Si Shen Decoction (SSD) treatment significantly reduces synovitis, ankle swelling, and bone erosion in RA, accompanied by decreased serum levels of CRP, TNFα, and IL-1β. The lead molecules listed from SSD are diosgenin, protodioscin, allantoin, pachymaran, 2β-hydroxybetulinic acid, alkaloids (nuciferine, dauricine, and liensinine), and flavonoids (quercetin, rutin, and kaempferol). SSD downregulates FABP4, IKKa, and p65 mRNA and protein expression, while increasing PPARγ, thereby inhibiting the FABP4/PPARγ/NFkB signalling pathway, which leads to reduced inflammation in RA [27]. Shu-Tong-Zhi-Yun Decoction (STZYD) alleviates RA symptoms by inhibiting TNF and NF-kB signaling pathways. STZYD regulates inflammation, immunity, and metabolism, including amino acid and arachidonic acid pathways. Bioinformatics and metabolomics analyses reveal potential biomarkers and mechanisms. Major active constituents known from the formula included quercetin, luteolin, and formononetin [28]. Recently, FHD was confirmed in a CIA rat model, and the anti-RA property of FHD was considered to be the control of autophagy and apoptosis of RA-FLSsz, besides the retarding of HIF-1α-mediated signaling and angiogenic processes. These combined actions show that it could be a multi-target drug for RA treatment. Qintengtongbi decoction (QTTBD) targets 175 RA-related genes, including AKT1, IL6, and TNF. Network pharmacology analysis reveals involvement in oxytocin and cytokine receptor signalling pathways. QTTBD’s mechanism of action in RA treatment is multifaceted, modulating inflammation and immunity [29].

Huang-Lian-Jie-Du decoction (HLJDD) treats RA by inhibiting immune-inflammatory responses. Key active ingredients, notably quercetin, target IL-17, TNF, and TLR signaling pathways. HLJDD alleviates synovial destruction and chemokine release. Quercetin’s anti-proliferative effects on RA-FLS validate HLJDD’s therapeutic potential. Other active constituents responsible for anti-RA activity from HLIDD are berberine, coptisine, epiberberine, palmatine, jatrorrhizine, baicalin, baicalein, wogonin, wogonoside, genipin, carotenoids, quercetin and rutin [30] Huangqi-Guizhi-Wuwu-Decoction (HGWD) mitigates RA by targeting various mechanisms and pathways. Its key active ingredients are quercetin, kaempferol, and beta-sitosterol, with its therapeutic benefits largely attributed to the TNF and IL-17 signaling pathways [31]. Wutou decoction (WTD) alleviates RA symptoms in rats by regulating metabolic pathways. Key components, including aconitine and quercetin, reverse the accumulation of abnormal metabolites. WTD reduces TNF-α and IL-6 levels, preventing synovial oedema, thus establishing a theoretical basis for clinical uses of WTD [32].

Jin Gu Lian Capsule (JGL) in TCM targets 52 drug targets in RA treatment, with 16 core active compounds, including flavonoids quercetin and salidroside. Other active components include anabasine, chlorogenic acid, protocatechuic acid, gaultherin, gaultheroside A, and flavonoid myricetin. Key therapeutic targets include IL1B, JUN, CXCL1, STAT1, and PTGS2, involved in immune-mediated inflammation. The therapeutic benefits of JGL are believed to be linked to its modulation of the IL-17/NF-κB signaling axis. Molecular docking analyses revealed strong binding of the main bioactive components of JGL to the key target proteins. The JGL therapy significantly decreased inflammation by inhibiting the IL-17/NF-κB signaling, lowering cytokine and chemokine levels, and downregulating the expression of inflammation-related genes [33]. Xinfeng Capsule alleviates RA by targeting AKT1 and TNF. Mairin, folic acid, cholesterol, and triptolide are key active compounds. XFC inhibits synovial cell proliferation and inflammation [34]. Jin-Wu-Jian-Gu Formulation (JWJGF) attenuates RA via IL33-ST2 signaling inhibition. Liquiritigenin is the key component; IL-33 is the core target. JWJGF inhibits RASF cell proliferation and alleviates RA symptoms [35].

The herbal formulations reviewed in this study were prepared from completely different plant materials, yet all network pharmacology investigations pointed mainly to the same few inflammatory pathways being involved, namely TNF, NF-kappaB, IL-17, and TLR-mediated ones. The flavonoids quercetin, kaempferol, luteolin, and berberine are identified more frequently as key compounds that are bioactive among several independent studies, indicating a possibility that these natural compounds might be major pharmacological targets that are responsible for the anti-inflammatory activity. Although the majority of studies have identified these similar signal transduction pathways using computational methods, only a few studies included molecular docking techniques and either in vitro or in vivo experiments to verify the biological relevance. As a result, the area of the transition from a predicted list of targets to biologically meaningful events remains quite large. So the research to follow has to make sure to carry out standard and reliable experimentations to get the actual biologically relevant mechanism out of the list of predicted targets.

### Cellular proliferation and apoptosis

RA features uncontrolled cellular proliferation and reduced apoptosis, which are mainly attributed to the abnormal regulation of intracellular signaling pathways. The activation of PI3K/Akt pathway not only leads to protein production but also prevents cells from dying, besides playing a role in the immune system. These attributes collectively contribute to synovial overgrowth and ongoing inflammation in RA. Likewise, the overuse of the JAK/STAT pathway escalates cytokine signaling by phosphorylating STAT and promoting its nuclear translocation. This ultimately leads to the production of inflammatory mediators and the growth of harmful immune cell types [36]. Similarly, the MAPK/ERK signaling cascade, initiated through MEK-mediated phosphorylation of ERK, regulates immune cell survival and pro-inflammatory gene expression. Its overactivation promotes synoviocyte proliferation and contributes to pannus formation in RA. The Ras/MEK/ERK axis also regulates immune cell proliferation and tissue remodelling. Hyperactivity in this pathway amplifies inflammatory feedback loops, worsening RA symptoms [37].

Triptophenolide (TRI) obtained from TCM, *Tripterygium Wilfordii*, demonstrated greater potential in managing RA by acting on diverse inflammatory signaling proteins, as indicated through integrative computational analyses. In murine models of RA, TRI alleviated joint inflammation and mitigated synovial damage by inhibiting pyroptosis. These findings suggest TRI could exert therapeutic effects via multiple biological pathways and may hold promise as a multi-target anti-inflammatory phytochemical [38]. *Porana sinensis* Hemsl. is a traditional medicinal herb with therapeutic effects on modulating mechanisms that control cell growth and death. Its main bioactive components, such as coumarins and chlorogenic acids, can reduce inflammatory reactions by decreasing the level of TLR2 and MyD88, leading to lower cytokine production, less oxidative stress, and alteration of PI3K/Akt, MAPK, NF-κB, and Toll-like receptor signaling pathways. These multiple-target effects have been shown to play a major role in protecting against RA-related inflammation and tissue injury by *Porana sinensis* Hemsl [39]. *Hedyotis diffusa* Willd (HDW) possesses anti-RA effects by inhibiting the proliferation of MH7A synoviocytes in a concentration- and time-dependent manner. RT-qPCR and Western blot analyses provide experimental evidence that these influences happen through the PI3K/Akt signaling pathway. HDW contains a wide range of bioactive compounds, namely iridoids, flavonoids, anthraquinones, triterpenoids, essential oils, and phenolic acids, which together are responsible for its anti-inflammatory, immunomodulatory, and antiproliferative effects via a multi-target mechanism of action [40].

*Arecoline hydrobromide* (AH) treats RA by inhibiting FLS proliferation, inducing apoptosis, and suppressing migration. AH targets the PI3K/AKT pathway and key proteins (CDK1, Bcl-2, vimentin) in synovial fibroblasts. In vivo studies confirm the efficacy of AH in relieving arthritis symptoms. Network pharmacology identifies F2, MAPK14, SRC, AKT1, and CTSK as potential direct targets of AH. The bioactive constituents in AH include mainly arecoline, arecaidine, guvacoline, guvacine and tannins. While studies suggest potential for RA treatment, more research is needed to understand its mechanisms and safely harness its therapeutic properties, especially considering the known toxicity of areca nut consumption [41]. Based on network pharmacology and lab experiments, it is apparent that *Odontites vulgaris* Moench can ameliorate inflammation by modulating the PI3K/Akt signaling pathway. The active molecules interfere with macrophage activation by down-regulating NO, TNF-α, IL-6, and IL-16 production, mechanisms that underlie its anti-rheumatic properties. This study elucidates the mechanism of action of *Odontites vulgaris* Moench and supports its therapeutic potential. The active constituents reported include iridoids (aucubin, catalpol, shanzhiside methyl ester and 8-epiloganic acid), flavonoids (apigenin, quercetin, apigenin-7-O-glucopyranoside and luteolin-7-O-glucopyranoside), phenolic compounds (acteoside, caffeic acid, and gastrodin [42].

*Paederia scandens* extract was identified to contain 27 active components that interact with key genes involved in RA development, including IL-1/3, PI3K, TNF, and JAK2. High-affinity binding between Paederia scandens extract and JAK2, a crucial component of the JAK-STAT signaling pathway, was observed. Based on results gathered in HFLS-RA cells, the extract of *Paederia scandens* has demonstrated anti-inflammatory and anti-proliferative effects by decreasing the levels of IL-6, IL-1β, and IL-17 and at the same time, it blocks the JAK2/STAT3 signaling pathway [43]. *Commiphora wightii* (C. wightii), a traditional Southeast Asian plant, shows potential in treating RA with no known cure. Network pharmacology and molecular docking reveal active compounds (Guggulsterol-V, Myrrhahnone B, Campesterol) targeting TNF, PIK3CA, and MAPK3 genes.C. wightii affects RA-related signaling pathways, exhibiting a preventive impact [44]. The *Paeoniae radix* rubra-*Angelicae sinensis* radix (P-A) drug pair treats RA by targeting the PI3K/AKT/NF-κB signaling pathway. Key compounds (caffeic acid, quercetin, paeoniflorin, baicalein) regulate PIK3CA, PIK3R1, AKT1, HSP90AA1, and IKBKB genes. RA model rats showed better synovial histopathology and relief of inflammation. The P-A drug pair has the potential to work against RA by regulating multiple targets [45].

Network pharmacology and molecular docking analyses revealed that *Saussurea involucrata* (SI) contains 27 bioactive compounds that together act on 665 genes, of which 119 are related to RA. Most importantly, the main targets were MAPK14, MAPK1, RELA, TNF, and MAPK8, which are very likely contributing to joint inflammation and destruction. Besides that, SI-based flavonoids such as xuelianlactone and involucratin strongly bind to these RA-associated targets, indicating the effectiveness of SI in the treatment of RA [46]. *Clematis Florida* (CF) exhibits anti-inflammatory and analgesic effects through MAPK/NF-kB pathway inhibition. Key targets include COX-2, TNF-α, and IL-6. CF alleviates arthritis symptoms in animal models, reducing inflammatory markers. Its natural compounds have therapeutic potential for the treatment of inflammatory diseases [47]. According to network pharmacology analyses, Tanshinone IIA (Tan IIA) from Salvia miltiorrhiza Bunge, works on the anti-RA effect by changing MAPK, AKT/mTOR, HIF-1, and NF-κB signaling pathways simultaneously. This ability to act on multiple targets limits the growth, movement, and spread of RA-FLSs and reduces inflammation and tissue destruction by inhibiting the production of pro-inflammatory mediators and matrix metalloproteinases [48]. *Juanbi-Tang* (JBT) treats RA via multiple absorbed compounds. JBT regulates MAPK, FoxO, and Rap1 pathways. 18 compounds and 17 metabolites contribute to its anti-RA effects [49]. The anti-rheumatic property of *Porana sinensis* Hemsl depends on five main bioactive compounds, which, in combination, influence 25 RA-related targets. By affecting the PI3K/Akt and HIF-1 signaling pathways, PSH inhibits the release of inflammatory mediators, lessens the production of cytokines, and is beneficial in the treatment of manifestations of RA. This paper presents the experimental data that justify the positioning of Porana sinensis Hemsl as a multi-target therapeutic agent for rheumatoid arthritis [50]. *Saposhnikovia divaricata* (SD; called “fangfeng” in China) exhibits anti-RA effects through regulating PI3K/AKT, IL-17, and apoptosis pathways. Prangenidin, a core ingredient, inhibits MH7A cell proliferation and induces apoptosis. In vivo studies confirm Prangenidin’s therapeutic effects in CIA mice. Findings provide insights into SD’s molecular mechanism and potential for RA treatment [51]. *Scutellaria doxy* treats RA by regulating PI3K/AKT and IL-17 pathways. Core ingredients (wogonin, prangenidin) exhibit anti-inflammatory/ immunomodulatory effects. Prangenidin inhibits MH7A cell viability and induces apoptosis [51].

Bavachinin’s (BVC) anti-RA activity has been linked to its ability to modulate several pathological processes, including synoviocyte proliferation, migration, apoptosis, and cytokine production. Experimental work revealed that BVC alleviates joint inflammation and tissue damage in CIA mice by modulating the PPARG/PI3K/Akt signaling pathway, highlighting its potential role as a multi-target therapy [52]. Network pharmacology studies have indicated that cinnamaldehyde (CA) may be protective against RA and osteoporosis through a multi-target, multi-pathway mechanism of action. CA is the regulator of 26 protein targets, which are involved in nitrogen metabolism, estrogen signaling, Rap1 signaling, and PI3K/Akt signaling pathways. In addition, the anti-inflammatory properties of cinnamaldehyde were demonstrated by experiments showing that the expression of several proteins associated with the diseases, such as EGFR, MAP2K1, MMP2, FGFR1, and MCL1, was decreased [53]. Emodin inhibits cell proliferation and induces apoptosis in RA cells by regulating the NF-κB pathway. Emodin’s anti-inflammatory effects are attributed to its capacity to inhibit NF-κB activation. In fact, emodin not only influences the expression of some of the most important genes that play a role in RA development, such as COX2, P38MAPK, and CASP3, but also multiple pathways it targets in this way could lead to relief of RA symptoms [54]. Peony’s total glucosides (TGP) exert their effect on RA through 20 possible targets, with the main mechanism of action being the inhibition of leukocyte recruitment and angiogenesis. The primary constituents, paeoniflorin (PF) and albiflorin (AF) form an interaction with the main targets like VEGFA, IL-6, and FGF1/2. Enriched pathways include VEGFR, interleukin signaling, and PI3K-Akt signaling. Molecular docking validates TGP’s potential as a multifaceted RA treatment [55]. Experimental studies reveal that Isopsoralen (IPRN) mitigates RA through several mechanisms of action, including cytokine production suppression, cell migration and invasion inhibition, and inflammatory signaling modulation. In CIA mice, IPRN reduced disease severity and prevented joint destruction. Targeting MIF, which is a key inflammatory mediator, is considered one of the main therapeutic effects of the drug; at the same time, it has anti-angiogenic properties [56].

Active compounds in Shentong Zhuyu decoction, such as quercetin, luteolin, and formononetin, have been shown to be bioactive. These compounds have been shown to target IL-2, IL-1α, IL-1β, and IL-6, which are highly associated with RA. Studies have also demonstrated that quercetin, luteolin, and formononetin can target anti-inflammatory cytokines, including IL-4 and IL-10, promoting an anti-inflammatory environment. The compounds of the traditional formula, Shentong Zhuyu Decoction, act by PI3K/AKT and NF-kB signaling and show anti-RA activity. They have been shown to modulate these pathways, potentially reducing inflammation and bone destruction [57]. Ba-Wei-Long-Zuan granule (BWLZ) exerts its anti-arthritic effect through the mTOR signaling pathway, regulating cell growth and metabolism. The steroid hormone biosynthesis pathway is also modulated, influencing immune response and inflammation. Additionally, BWLZ affects amino acid metabolism and ketone body synthesis, further contributing to its therapeutic effects. By targeting these pathways, BWLZ offers a promising approach to treating RA [58]. QufengZhitong Capsule (QFZTC) alleviates RA by inhibiting the inflammatory response. Key targets include IL6, IL1B, VEGFA, JUN, and TNF. Luteolin, Kaempferol, and Myricetin exhibit strong affinity with TNF. QFZTC’s anti-RA effects are mediated through the MAPK pathway regulation [59].

Network pharmacology analysis identifies the Ras/MEK/ERK signaling pathway as a key mechanism for Baihu Jia Guizhi Decoction (BHJGZ) in RA. In adjuvant arthritis rat models, BHJGZ significantly lowers Ras protein levels and inhibits Ras/MEK/ERK pathway activation. Using Ras and MEK inhibitors, lonafarnib and mirdametinib, further decreases phosphorylated ERK and pro-inflammatory factors IL-6 and TNF-α. Molecular docking reveals strong interactions between BHJGZ compounds and target proteins in this pathway. BHJGZ reduces paw swelling, joint inflammation, and improves synovial histopathology in RA models, highlighting its anti-rheumatic effects through Ras/MEK/ERK pathway inhibition [60]. Huangqi-Guizhi-Wuwu-Decoction alleviates RA through the key response components group (KRCG). KRCG’s 59 components target cAMP, PI3K-Akt, and HIF-1 alpha pathways. Four key components synergistically inhibit the inflammatory response [25]. Qin Xi Tong (QXT ) treats RA and osteoarthritis via IL-17, MAPK, and TNF signaling inhibition. 161 potential targets and 12 core targets identified through network analysis. QXT regulates joint inflammation and injury progression [61].

The‍‌‍‍‌ collection of these studies collectively depicts a largely harmonized scenario where the role of the PI3K/Akt pathway is essentially the main driver in synoviocyte proliferation and apoptosis in RA. The effects of different herbal medicines on a number of the upstream molecules have been documented, with at least some of the studies having also looked into the downstream effect of these herbs on the signaling pathways PI3K/Akt, MAPK, and JAK/STAT, amongst others. Therefore, that all of them eventually converge onto the same biological outcome may suggest one of the main aims of treatment is to control the survival of fibroblast-like synoviocyte. The other hand, however, the variation in proposed molecular targets and models of experimental validation is quite significant; so, a direct comparison between the various studies is ‍‌‍‍‌challenging.

### Angiogenesis and metabolism regulation

Dysregulation of angiogenesis and metabolic pathways contributes to continuous inflammation and tissue damage in RA. HIF-1 (Hypoxia-inducible factor-1) acts as the major factor promoting VEGF-dependent angiogenesis, the release of the inflammatory cytokines, immune modulation, and the proliferation of the inflammatory cells, thus speeding up the progression of the disease in RA with the help of the hypoxic microenvironment [62]. The VEGF signaling pathway is fundamental to the process of new blood vessel formation (angiogenesis). It is also strongly involved in inflammation, as it stimulates endothelial cell activity and increases blood vessel permeability. When this pathway is poorly regulated, it is a key feature of chronic inflammatory disorders and cancers and a major factor in their progression [63]. The mTOR (mechanistic target of rapamycin) pathway is a central regulator of cell growth, proliferation, and metabolism. It scans signals from nutrients, growth factors, and energy status to regulate protein synthesis and autophagy. Besides, mTOR is a player in inflammation, modulating immune responses and affecting the function of both innate and adaptive immune cells. Deregulation of mTOR is also the cause of chronic inflammation and autoimmune diseases [64]. Metabolic pathways responsible for amino acid and lipid biosynthesis are very important as they supply the essential components for cell growth and proliferation. During inflammation, immune cells alter their metabolism, leading to increased production of lipids and amino acids that help maintain their function. Pro-inflammatory cytokines TNF-α and IL-6 can alter lipid metabolism, leading to increased free fatty acids and triglycerides, which, in turn, exacerbate inflammation. As a result, focusing on these metabolic pathways may be a useful tactic for lowering inflammation and treating associated conditions [65].

Total saponins of *Panax japonicus* C.A. Meyer (TSPJs) inhibit RA-related angiogenesis by targeting HIF-1 and VEGF pathways, suppressing tube formation in EA. hy926 cells. TSPJs bind to HIF-1α, VEGFA, and ANG-1, alleviating CIA symptoms and reducing inflammation, angiogenesis, and joint damage. TSPJs decrease the number of vessels and CD31 expression, as well as HIF-1α, VEGFA, and ANG-1 levels in serum and synovial tissues. This mechanistic insight highlights TSPJs’ potential as an anti-angiogenic and anti-inflammatory agent for RA treatment [66]. Differential analysis revealed 2670 differentially expressed genes (DEGs) and four active components, based on predictions of 371 drug targets, with 52 genes overlapping in the PPI network and in KEGG analysis. KEGG enrichment showed angiogenesis-related pathways such as VEGF and HIF-1 signaling. Correlation analysis identified the main targets, SRC and STAT3, with significant pathway correlations. Total saponins from Panax japonicus treatment restrained CIA manifestations in mice and reduced VEGFA, HIF-1a, IL-13, and IL-17 A expression, thus highlighting its potential in RA [67].

While a number of works have focused on inflammatory signaling in RA from a network pharmacology standpoint, only a limited number of studies have used this platform to dissect the mechanisms of angiogenesis and metabolic reprogramming. Nonetheless, there is a consensus among these studies that HIF-1, VEGF, and mTOR are the most important regulatory nodes. In view of this finding, it is likely that future network pharmacology studies will be directed more and more towards metabolic remodeling and hypoxia, considering that these are the new disease-driving processes rather than only secondary effects of the disease pathology.

### Oxidative stress and mitochondrial function

Oxidative stress and mitochondrial dysfunction significantly contribute to inflammation. AMP-activated protein kinase (AMPK) is key in cellular energy balance, promoting mitochondrial growth and function while facilitating the removal of damaged mitochondria through mitophagy. This process helps reduce oxidative stress and inflammation, making AMPK a promising target for treating inflammatory disorders [68]. One component of the innate immune system, the NLRP3 inflammasome, is responsible for activating pro-inflammatory cytokines such as IL-1β and IL-18. Nonetheless, when mitochondria fail to function properly, they can inadvertently trigger NLRP3, leading to sustained inflammation. Therefore, intervention in the NLRP3 inflammasome is being considered as one of the possible ways of treating different inflammatory disorders [69]. In cells, mitochondria are the main sources of ROS generation. Although these species are important for signaling-mediated cellular functions, they can also cause oxidative stress and cellular injury when produced in excess. To avoid such a situation, it is necessary to balance mitochondrial function through mechanisms such as mitophagy and antioxidant defence, thereby controlling ROS levels. Restoring mitochondrial function and limiting oxidative stress are highly effective ways of dealing with inflammation and preventing the diseases related to it [70].

Osthole (OST) relieves RA symptoms by activating AMPK, inhibiting the NLRP3 inflammasome, and restoring mitochondrial homeostasis. OST prevents the secretion of inflammatory factors, induces apoptosis, and reduces ROS production in FLS cells. Animal experiments have also shown that OST can effectively treat CIA in rats by improving joint defects and inhibiting synovial inflammation. OST may have further therapeutic potential for RA [69]. Notopterygium can relieve RA symptoms by controlling the activation pathways of the NLRP3 inflammasome. Notopterygium extract (NE), has anti-inflammatory activity both in AA rats and in LPS-stimulated RAW264.7 cells. The ability to modulate mitochondria is the main mechanism underlying NE’s anti-inflammatory properties [71].

What is intriguing is that oxidative stress pathways are highly interconnected with inflammatory signaling. This is mainly because ROS generation, Nrf2 activation, and mitochondrial dysfunction are directly linked to, or result in, the activation of inflammatory factors (NF-κB and MAPK). In this sense, plant compounds that can act as dual agents to suppress oxidative stress and inflammation might offer better therapeutic options than compounds targeting only one of these processes.

### Bone and joint health pathways

Osteoclasts, which are cells that specifically break down bone tissue, are mainly regulated by RANKL and M-CSF factors that are responsible for their differentiation. Pro-inflammatory cytokines such as IL-1 and TNF-α can increase osteoclast activity, leading to excessive bone resorption and inflammatory bone diseases such as RA. The strict control of this pathway is important for the maintenance of bone balance, and a problem in this control can cause bone loss and inflammation [72]. The Wnt/β-catenin signaling pathway plays an important role in bone generation and remodeling; it is triggered when Wnt proteins bind to Frizzled, a family of G-protein-coupled receptor proteins, which causes β-catenin to build up and move into the nucleus. Once inside the nucleus, β-catenin modulates the expression of genes necessary for the maturation of osteoblasts. Nevertheless, inflammatory cytokines can suppress this pathway, resulting in reduced bone formation and skeletal deterioration. In the same vein, the Sonic hedgehog (Shh) pathway is indispensable for skeletal morphogenesis and bone healing. Upon Shh binding to Patched (PTCH), Smoothened (SMO) is activated, which in turn affects the proliferation and maturation of chondrocytes and osteoblasts. Inflammation leads to abnormalities in Shh signaling that lengthen bone damage. Moreover, inflammation worsens chronic inflammatory diseases [73].

Platycodin D (PD) has potential therapeutic effects in RA by inhibiting synovial proliferation and inflammation. It initiates cell death, changes mitochondrial membrane potential, and controls Sonic hedgehog signaling. It down-regulates pro-inflammatory cytokines (TNF-α, IL-6) and restores joint functions in CIA rats. PD could be a new drug to treat RA. Research is needed to confirm the finding [74]. Huangqin Qingre Chubi Capsules (HQC) help treat depression in patients with RA (RA-dep) by both reducing inflammation and increasing neurotransmitters. HQC specifically affects the Wnt/β-catenin signaling pathway, as it not only binds Wnt1 to but also activates the signaling. This effect contributes to reduced hippocampal damage and lessens depressive symptoms. HQC has the potential to be an effective drug for RA-dep treatment [75].

It should also be emphasized that several studies have reported various signaling molecules. Nevertheless, the majority identified RANKL/OPG, Wnt/β-catenin, MMPs, and osteoclast differentiation as common pathways mediating bone destruction. This suggests that herbal treatments, besides preventing and inhibiting inflammatory mediators, are also able to modulate the activities of cells, leading to the restoration of tissues, especially in bones, through the regulation of bone remodeling processes.

### Specialized pathway

Maintaining immune balance highly depends on the interplay between regulatory T cells (Tregs) and T helper 17 cells (Th17). Tregs have suppressive functions, which are essential for inducing tolerance and limiting immune responses. On the other hand, Th17 cells initiate inflammation. A shift in this fine equilibrium towards increased Th17 activity and reduced Treg function leads to autoimmune disorders and persistent inflammation. Anti-inflammatory therapies aim at reestablishing the equilibrium, thus restoring the immune system to its original state [76]. Besides, p53, a protein that suppresses tumors, plays a bigger part in managing the cell cycle, leading to cell death, and changing inflammation. Specifically, p53 controls the making of inflammatory cytokines and is the main player through which cells react to stress. p53 that is turned on, functions as a signal for stopping inflammation by causing apoptosis of the damaged cells and lowering the amounts of pro-inflammatory substances, which leads to healing and getting back to the usual condition [77]. The CCR5 signaling pathway is an essential player in the regulation of inflammation as it mediates the migration and activation of immune cells. CCR5, a chemokine receptor, primarily binds three ligands: CCL5, CCL3, and CCL4. The binding of these ligands triggers a series of intracellular alterations culminating in the recruitment of several immune cells, such as T lymphocytes, macrophages, and dendritic cells, to the sites of inflammation, which eventually enhances the immune response [78].

Duanteng Yimu Tang (DTYMT) can treat rheumatoid arthritis (RA) through modulating the Treg/Th17 ratio. It has been verified that DTYMT inhibits Th17 cell differentiation, promotes Treg cell generation, and alters cytokine levels. As a result, joint destruction and inflammation in RA are lessened. DTYMT can be an effective agent for treating RA [79]. The sesquiterpene isolated from *Artemisia lavandulaefolia DC.*, 5a-Hydroxycostic acid, has shown potential for the treatment of RA. It induces the expression of the Androgen Receptor (AR), indicating a new molecular mechanism [80]. Xiaoyao-Qingluoyin (XYQLY) and Qingluoyin (QLY) both relieve RA symptoms in rats. XYQLY is more effective in reducing the inflammation, LPS signaling, and Th17 cell numbers. It is also capable of modulating innate immunity, leading to an immune improvement. XYQLY has a stronger anti-rheumatic effect and warrants further clinical testing [81]. The anti-RA mechanism of Hedyotis diffusa Willd (HDW) is based on the modulation of the MMP9/miR-204-5p/MIAT axis. The work, through a blend of bioinformatics and biological experiments, unveiled that HDW inhibits RA-FLS proliferation and MMP9 expression. This study is the first one to investigate the ceRNA mechanism of traditional Chinese medicine in the treatment of RA [82].

Taken altogether, these studies imply that the specialized immune-regulatory pathways that are involved in the disease may be new therapeutic targets that are more than the typical inflammatory signaling pathways that are known to contribute to RA. In contrast to the well-known pathways such as NF-κB, PI3K/Akt, and MAPK, there are other mechanisms that involve Treg/Th17 homeostasis, p53-mediated immune regulation, androgen receptor signaling, and ceRNA networks, which can offer more understanding of why herbal medicines have effects on the immune system. Still, the number of studies supporting the specialized pathways seems very small, as most of the results come from individual experimental studies and need to be validated in another lab. As a result, further research that combines network pharmacology with transcriptomics, single-cell omics, and functional experimental studies could be an effective way to demonstrate that the new molecular mechanisms are truly helpful in therapy and how far they can be applied to the problem of RA.

### Multi-component and multi-target pathway

In-depth analyses of Compound Ruteng (CRT) reveal 92 active compounds that interact with 391 proteins, thereby relieving RA symptoms. Biological tests and X-ray images show that inflammation and joint damage are reduced, and cytokine markers are less expressed. CRT is a candidate for being a drug for RA treatment [83]. Emodin’s (EMO’s) anti-RA activity is associated with 12 key targets, influencing monocyte function and inhibiting inflammation. Studies carried out both in vitro and in vivo demonstrate that EMO is capable of blocking cell differentiation, diminishing IL-6/IL-13 expression, and altering COX2/HMGB1/p38 signaling. Sequencing of synovial fibroblasts confirms EMO’s anti-inflammatory properties. EMO could be a new drug for RA treatment [84]. Naringin reduces RA inflammation and MMPs production and also enhances RA cells’ apoptosis through the PI3K/Akt and MAPK/ERK signaling [85].

Experiments conducted in vitro demonstrated that these compounds can inhibit the cell division, movement, and invasion of RA-FLS, and also lead to the decrease of NF-KB signaling. By applying network pharmacology and molecular docking techniques, the researchers discovered that the compounds impact numerous targets, especially tyrosine kinases, to counteract RA. According to this study, umbelliferone and scopoletin are the anti-rheumatic substances, and the molecular pathways through which they carry out their functions include the suppression of tyrosine kinases and the blocking of NF-kB in the RA-FLSs [86].

Jishe Qushi capsules (JSQS) demonstrated potent efficacy in treating RA through a combination of multi-component synergies (7 major bioactive components), multipathway modulation (cancer, PI3K/AKT, MAPK, AhR), multi-target effects (118 anti-RA targets, including TP53, IL6, VEGFA), and synergistic actions. Seven bioactive compounds regulate 118 anti-RA targets, including TP53 and IL6. In addition, clinical trials have demonstrated significant alleviation of symptoms, reduced inflammation, and improved quality of life. JSQS decelerates RA development, thereby demonstrating its potential as a supplement to conventional therapeutic strategies [87]. “Miao-Fu-Zhi-Tong (MFZT) granules,” an ethnic herbal prescription, were analysed using UHPLC-MS/MS coupled with network pharmacology. 207 chemical species were found, including rare guanidines, and 84 active compounds potentially targeting NF-κB, NOD-like, and RA signaling cascades were identified. A quantitative technique was formulated for 29 efficacious substances, with berberine and others serving as chemical markers. The present work provides a solid foundation for MFZT’s anti-gout effects and quality control from a chemical perspective [88]. Fuzi decoction (FZD) is a potential anti-osteoporosis agent that acts by inhibiting osteoclast formation. The bioactive compounds present in it mostly perform their functions through the regulation of the NF-κB signaling pathway, which leads to the suppression of pro-apoptotic factors. Both in vivo and in vitro studies have shown that FZD can function as a therapeutic. More studies are needed to determine the clinical applications of FZD [89].

Wutou decoction works by targeting a specific pathway (PI3K-AKT-mTOR-HIF-1α) that contributes to angiogenesis in RA. This pathway is involved in the growth and migration of cells that line the blood vessels [90]. *Rhizoma Coptidis* exhibits anti-inflammatory effects by inhibiting glycolysis and modulating gut microbiota. The PI3K-AKT and HIF-1α pathways play crucial roles in regulating glycolysis. Firmicutes and Bacteroidetes gut microbiota contribute to RA pathology. The therapeutic potential of *Rhizoma Coptidis* in RA is supported by these findings [91]. *Achyranthes bidentata* Blume saponins (ABS), which may be a new RA therapy, undergo drug-metabolising mechanisms such as hydroxylation, hydrogenation, and oxygenation. Network pharmacology study shows that ABS can target MAPK, PI3K-AKT, as well as p53 signaling pathways, and therefore may exert its therapeutic effects through these pathways. In a dose-dependent way, ABS improves the symptoms of RA by restoring the balance between pro- and anti-inflammatory factors, as well as by reducing ERK phosphorylation in MAPK signaling [92].

Spirulina acts on RA through the inhibition of key protein targets such as NFKB, NLRP3, TNF, MMP-9, TLR4, and VEGFA and main pathways like PI3K-Akt, MAPK, osteoclast differentiation, and VEGF signaling. Spirulina reduces inflammatory factors like IL-1, IL-6, IL-17, TNF-α, MMP-9 and influences RA-related proteins (TLR4, NLRP3, VEGF, caspases, NF-KB p65). This research demonstrates Spirluna’s mode of action at the molecular level and its effectiveness against RA, thereby Posing it as a potential therapeutic agent [93]. ErshiwuweiLvxue Pill alleviates RA through multi-component, multi-target mechanisms. 22 bioactive compounds regulate PI3K-Akt, JAK-STAT, MAPK, and TNF pathways. Ellagic acid, quercetin, and kaempferol show high affinity with PTGS2, STAT3, and VEGFA targets [94].

Flowers of Rhododendron molle G. Don (RMF) decrease the symptoms of RA by blocking the production of pro-inflammatory cytokines (IL-1β, IL-6, TNF-α) and attenuating bone loss in CIA rats. Four major targets (AKR1B1, TPH1, CYP1A1, CYP1A2) and their metabolites function in steroid hormone biosynthesis, tryptophan metabolism, and galactose metabolism. Five components (Rhodojaponin-II, Rj-II, Rj-V, Rj-VI, quercetin) play a role in RMF’s anti-RA properties. RMF’s healing impact on RA is due to its ability to change metabolic pathways and suppress pro-inflammatory factors [95].

The studies suggest that the effectiveness of herbal medications against RA stems from the interaction of multiple active ingredients that act on interconnected molecular pathways. Herbal formulations can differ greatly from one another, but network pharmacology almost always identifies common regulatory mechanisms, such as NF-κB, PI3K/Akt, MAPK, JAK/STAT, and HIF-1α pathways. The research shows that phytochemicals, though structurally and functionally diverse, converge on shared pathological networks in RA. Although there are experiments and molecular dockings that support these results, there are still difficulties associated with pharmacokinetics, standardization of formulations and lack of proper clinical trials. It is therefore important in future work to use methods such as network pharmacology and multi-omics technologies as part of a comprehensive approach to develop robust evidence to support interventions for RA.

In summary, traditional medicines can be explored in drug discovery to identify the lead using network pharmacology approaches. A significant trend identified through the analysed network pharmacology papers is the repetition in the identification of a small set of phytochemicals, mainly quercetin, kaempferol, luteolin, berberine, celastrol, and triptolide, among others, from quite different herbal preparations in terms of chemistry. Their reappearance across various research articles suggests they are core therapeutic agents rather than merely common components. Quercetin, one of the most commonly reported flavonoids, has also been shown to modulate inflammatory mediators such as TNF, IL-6, PTGS2, AKT1, MAPKs, and RELA, primarily by regulating the NF-κB, PI3K/Akt, MAPK, and JAK/STAT pathways. Kaempferol shares these molecular targets but appears more pronounced in regulating osteoclast differentiation, oxidative stress, and bone remodelling, suggesting an additional role in preserving joint health. Similarly, luteolin has been associated with the suppression of pro-inflammatory cytokines, macrophage activation, and Th17-type immune response, indicating its potential to control immune hyperactivity during RA development. By comparison, compounds like berberine show a wider spectrum of effects by not only regulating inflammatory signals but also cellular metabolism and gut microbiota homeostasis. On the other hand, diterpenoids such as celastrol and triptolide have been shown to possess potent immunosuppressive and anti-inflammatory effects; however, they come with certain limitations, e.g., potential toxicity and concerns about therapeutic safety. This line of thinking suggests that, even though phytochemicals tend to appear consistently in network pharmacology analyses, their contributions are quite diverse and therefore not redundant. The fact that such pharmacologically active constituents would all appear simultaneously in conventional herbal formulas could mean that, by means of simultaneously targeting several related biochemical processes, including inflammation, modulation of the immune system, blood vessel formation, production of oxygen radicals, and bone resorption, the overall treatment effectiveness is increased due to synergies among constituents. Nevertheless, there are few direct experimental studies that verify synergies among the phytochemicals in question, since most of the literature relies on computational methods such as target prediction and molecular docking, while very few studies focus on combination or pharmacokinetic evaluation. Table 1 provides a side-by-side review of the commonly encountered phytochemicals, their plant sources, main molecular targets, signaling pathways, pharmacological activities, and supporting evidence, showing not only the highlights but also, what’s lacking in the area of network pharmacology-guided herbal drug discovery for RA.

**Table 1:** Comparative bioactivity of key phytochemicals uncovered by network pharmacology to treat RA

Lead Molecule	Source or Formulation	Major Drug Targets	Modulated Pathways	Major Therapeutic Effects	Experimental validation	Reference
Quercetin	HLJDD, EMS, GSZD, HGWD	TNF, IL6, PTGS2, VEGFA	NF-kB, IL-17, TNF	Anti-inflammatory, anti-proliferative	In Silico, In vitro, In vivo	[26, 30, 31]
Kaempferol	Tripterygii Radix, GSZD	AKT1, TNF, JUN	PI3K/Akt, TNF	Immune modulation, apoptosis regulation	In Silico, In vitro, Tested in Human cells	[17]
Luteolin	QLY, QFZTC, LR	AKT1, IL6, TNF	PI3K/Akt, MAPK	Anti-inflammatory, anti-migratory	In Silico, In vitro, in vivo	[59, 81]
Celastrol	Tripterygium hypoglaucum	PTGS2, TNF, RELA	TNF/NF-kB	Cytokine suppression	In silico, In vitro	[19]
Triptolide	Tripterygium wilfordii	TNF, CASP3, IL6	TNF, NF-kB	Synovial inflammation inhibition	In silico, in vitro, In vivo	[34, 38]
Berberine	HLJDD, EMS	IL6, PTGS2	IL-17, TLR	Anti-inflammatory activity	In Silico, In vitro, In vivo	[26, 30]
Paeoniflorin	P-A drug pair, TGP	PIK3CA, AKT1	PI3K/Akt/NF-kB	Reduced synovial inflammation	In silico, in vitro, In Vivo	[45, 55]
Formononetin	STZYD, HQGZ	IL6, TNF	PI3K/Akt, NF-kB	Immunomodulatory activity	In silico, In vitro, In vivo	[28, 96]
Wogonin	EMS, Scutellaria doxy	IL6, STAT3	IL-17, PI3K/Akt	Anti-inflammatory effects	In silico, In vitro, In vivo	[26, 51]
Liquiritigenin	JWJGF	IL-33	IL33-ST2	Reduced RA-FLS proliferation	In silico, In vitro, In vivo	[35]
Bavachinin	Fructus Psoraleae	PPARG, AKT1	PPARG/PI3K/Akt	Anti-proliferative effects	In silico, In vitro, In vivo	[52]
Emodin	Rheum species	COX2, CASP3, MAPK	NF-kB, p38 MAPK	Apoptosis induction	In silico, In vitro	[54, 84]
Tanshinone IIA	Salvia miltiorrhiza	MAPK1, AKT, HIF-1α	MAPK, mTOR, HIF-1	Inhibition of RA-FLS proliferation	In silico, In vitro, In vivo	[48]
Naringin	Citrus species	AKT, ERK	PI3K/Akt, MAPK/ERK	Anti-inflammatory and pro-apoptotic	In silico, In vitro, In vivo	[85]
Umbelliferone	Saussurea laniceps	Tyrosine kinases, NF-kB	NF-kB	Reduced RA-FLS migration	In silico, In vitro, In vivo	[86]
Scopoletin	Saussurea laniceps	Tyrosine kinases	NF-kB	Anti-inflammatory activity	In silico, In vitro, In vivo	[86]
Arecoline	Areca catechu	AKT1, MAPK14	PI3K/Akt	Inhibition of synovial proliferation	In silico, In vitro, In vivo	[41]
Diosgenin	SSD	PPARγ, NF-kB	FABP4/PPARγ/NF-kB	Reduced inflammation	In silico, In vitro, In vivo	[27]
Calycosin	Astragalus membranaceus	JNK, NF-kB	JNK/NF-kB	Macrophage regulation	In silico, In vitro	[24]
Isopsoralen	Psoralea corylifolia	MIF	Angiogenesis pathways	Anti-angiogenic effects	In silico, In vitro	[56]
Aconitine	Wutou decoction	HIF-1α, TNF	PI3K/Akt/mTOR/HIF-1α	Reduced synovial edema	In silico, In vitro, In vivo	[90]
Ellagic acid	ErshiwuweiLvxue Pill	PTGS2, STAT3	PI3K/Akt, JAK/STAT	Anti-inflammatory effects	In silico, In vitro, In vivo	[94]

## Limitations of network pharmacology approaches

On the one hand, network pharmacology provides a holistic framework that enables the dissection of complex herbal drugs at the molecular level and can be used to predict their multitarget mechanisms. On the other hand, their practical application is hampered by a lack of appropriate methodologies for handling large data sets, a shortage of computing power for big data analysis, and limited experimental confirmation at the molecular level. The most important limitation is database heterogeneity and variability [97]. Public repositories that provide queries for phytochemical ingredients and disease targets exhibit significant inconsistencies in their curation standards, annotation depth, and update frequency. Different databases, such as TCMSP, PubChem, DrugBank, and GeneCards, do not agree on which compounds interact with which targets because their data vary from one source to the next. This results in compound-target predictions that are often contradictory, many false-positive results, and many plant-unique metabolites that either go completely unnoticed or are lumped together. In addition, platforms that have not been updated regularly introduce serious biases that generate noise, interfering with network topology and pathway enrichment analyses.

Almost all computational techniques for target fishing rely on structural matching or ligand-protein similarity. These approaches, however, are mostly static and cannot account for changes in cellular environments or other dynamic aspects of biology. One significant drawback of these methods is that they provide binary outputs (bound/unbound) only, with no information about the actual biological effect: for example, whether a drug compound is acting as an agonist, antagonist, or an allosteric modulator. Furthermore, many of these predictive computational models are very poor at predicting the specific binding affinities of a compound (*K*_*d*_, *K*_*i*_, *or IC*_*50*_) at intracellular concentrations under physiological conditions, which can make it very difficult to distinguish truly druggable targets from off-target effects due to low binding affinity [8]. Routine in silico screening involves the use of very strict ADME (absorption, distribution, metabolism, excretion) filters, for example, oral bioavailability and drug-likeness, which, in effect, greatly simplify human physiology. Such parameters consistently overlook fundamental in vivo biotransformations, including hepatic phase I/II metabolism, multi-herb synergistic transport, and gut microbiota-mediated catabolism. As a result, computer models regularly rank the mother phytochemicals for their potential, even though these have never reached the circulation at effective doses, and they have no idea about the active, bioavailable secondary metabolites, which are the real sources of therapeutic effect in the clinic [7].

Many current network pharmacology publications still rely solely on computational methods. Without experimental validation, these papers present computer-generated biochemical network results as definitive biological proofs. The rules clearly state that network predictions must be validated against methods such as biophysical binding assays (e.g., SPOTs or calorimetry-based assays), multiple OMNIS datasets, and real cell or animal disease models to confirm protein interactions and pathway changes [7]. Natural variations in factors such as botanical source localization, time of harvest, various preparation procedures like milling and grinding, and different extraction methods are the major contributors to the chemical diversity of one batch of herbal drugs compared with another. If the chemical composition of herbal products is not controlled to an extent that can be verified chemically by methods such as HPLC or LC-MS/MS fingerprinting to determine the quantity of the most active ingredient, the computational models will be based solely on ideal chemical profile data [98].

## Conclusion and future perspectives

The growing integration of network pharmacology with bioinformatics, metabolomics, molecular docking, and experimental validation has substantially expanded current understanding of herbal therapeutics in rheumatoid arthritis. Rather than acting through isolated molecular targets, traditional medicinal formulations appear to influence interconnected signaling networks associated with inflammation, immune dysregulation, oxidative stress, angiogenesis, and synovial hyperplasia. This review’s summary highlights the critical role of systems-level methods in interrogating herbal medicines: identifying active plant compounds, understanding their direct and off-target interactions at the molecular level, and deciphering the pharmacology of multi-component herbal formulations through synergistic mechanisms. Phytochemicals such as quercetin, kaempferol, berberine, triptolide, and luteolin are able to potently modulate the immune system in a wide variety of ways due to their ability to concurrently adjust the activity of numerous intracellular pathways, such as those mediated by NF-κB, PI3K/Akt, MAPK, and JAK/STAT signaling chain. Regardless of their distinct plant sources and chemical compositions, these phytoconstituents collectively influence critical inflammatory molecules such as TNF, IL-6, AKT1, RELA, PTGS2, and MAPKs. Consequently, this serves as evidence that different herbal remedies converge on the same molecular pathways implicated in RA. Notwithstanding these developments, several difficulties remain unaddressed, such as the heterogeneity of herbal ingredients, the lack of a unified pharmacokinetic framework, limited clinical validation, and reliance solely on computational predictions. Subsequent work employing artificial intelligence (AI), multi-omics platforms, systems biology, and well-controlled clinical trials could improve translational accuracy and lead to the development of precision phytomedicine approaches for the treatment of rheumatoid arthritis. The integration of AI, multi-omics, and systems biology is revolutionizing network pharmacology in herbal drug discovery for RA. Instead of viewing herbal formulations as static mixtures, advanced algorithms analyze complex interactomes to identify bioactive synergies and predict biological alterations. Multi-omics approaches provide detailed molecular profiles of the synovial microenvironment, capturing dynamic interactions between host and herbs. This synergy facilitates precision phytomedicine, enabling tailored, multi-target herbal therapies based on specific patient dysregulations. By linking computational predictions with experimental assays and clinical biomarkers, we can enhance the development of standardized, mechanism-driven natural therapies for RA. Collectively, network pharmacology represents a valuable bridge between traditional medicinal knowledge and modern systems therapeutics, supporting the discovery of evidence-based multi-target interventions for chronic inflammatory disorders.

## Data Availability

No datasets were generated or analysed during the current study.
